# Effect of health systems context on infant and child mortality in sub-Saharan Africa from 1995 to 2015, a longitudinal cohort analysis

**DOI:** 10.1038/s41598-021-95886-8

**Published:** 2021-08-11

**Authors:** Ryan A. Simmons, Rebecca Anthopolos, Wendy Prudhomme O’Meara

**Affiliations:** 1 Duke Global Health Institute, Box 90519, Durham, NC 27708 USA; 2 Department of Biostatistics, Duke University School of Medicine, Durham, NC 27708 USA; 3 Department of Biostatistics, Columbia University Mailman School of Public Health, New York, NY USA; 4 Department of Medicine, Duke University School of Medicine, Durham, NC 27708 USA

**Keywords:** Health services, Public health

## Abstract

Each year, > 3 million children die in sub-Saharan Africa before their fifth birthday. Most deaths are preventable or avoidable through interventions delivered in the primary healthcare system. However, evidence regarding the impact of health system characteristics on child survival is sparse. We assembled a retrospective cohort of > 250,000 children in seven countries in sub-Saharan Africa. We described their health service context at the subnational level using standardized surveys and employed parametric survival models to estimate the effect of three major domains of health services—quality, access, and cost—on infant and child survival, after adjusting for child, maternal, and household characteristics. Between 1995 and 2015 we observed 13,629 deaths in infants and 5149 in children. In fully-adjusted models, the largest effect sizes were related to fees for services. Immunization fees were correlated with poor child survival (HR = 1.20, 95% CI 1.12–1.28) while delivery fees were correlated with poor infant survival (HR = 1.11, 95% CI 1.01–1.21). Accessibility of facilities and greater concentrations of private facilities were associated with improved infant and child survival. The proportion of facilities with a doctor was correlated with increased risk of death in children and infants. We quantify the impact of health service environment on survival up to five years of age. Reducing health care costs and improving the accessibility of health facilities should remain a priority for improving infant and child survival. In the absence of these fundamental investments, more specialized interventions may not achieve their desired impact.

## Introduction

In 2015, the international community embraced the Sustainable Development Goals, which include the ambitious goal of eliminating preventable child deaths by 2030. Annually, more than 50% of childhood deaths occur in sub-Saharan Africa^1,2^. Thirty-four percent of these deaths occur in the neonatal period^3^ and of these 70% are preventable through effective prenatal care or safe delivery^4,5^. After the neonatal period, treatable or preventable infectious diseases such as pneumonia, malaria, and diarrhoea, account for between 64 and 90% of childhood deaths^3,6^. In short, a large proportion of deaths between birth and the fifth birthday are a result of common causes that are avoidable through affordable and effective interventions. Therefore, child mortality remains unacceptably high where these causes dominate.

Health facilities are the primary distribution channel for many cost-effective interventions such as immunizations, safe delivery and insecticide-treated bednets. Although each individual intervention has great potential to reduce morbidity and mortality, the population-level impact of these life-saving interventions may be limited by the health systems that deliver them^4,7^. For example, an estimated 15–29% of people in SSA live more than 2 h travel time from the nearest public hospital ^8,9^ which highlights the limitation of geographic distribution of health services. In an era of renewed emphasis on Universal Health Care (UHC), the importance of measuring health system coverage and quality is being recognized^10–12^. Previous studies in sub-Saharan Africa have assessed quality by benchmarking on guidelines ^13,14^. However, very few studies have extended these measures to understand their relationship to health outcomes^15^.

Inadequate health systems may be impeding progress in child survival, but rigorous evaluation of delivery strategies is methodologically challenging^7,16,17^. Here we adopt the perspective of Watkins^18^ and focus on the ‘platforms required to deliver essential interventions’ as a critical factor in the success of UHC. Specifically, with respect to the Countdown to 2015 framework^19^, we focus on three key health system building blocks—infrastructure, workforce, and financing—which, when taken from the point of view of the user, become *access*, *quality* and *cost*. The objective of this study is to better understand the impact of the health system as a delivery platform*,* independent of specific interventions, on child survival. We focus on subnational measures of health system context motivated by the observation that child mortality varies more within countries than between countries^20^. We assembled a longitudinal cohort of children born in seven countries over 20 years and estimate the impact of their local health services context on the risk of death in infancy and early childhood. We divide the health context into three dimensions—access, quality and cost—and individually investigate their relationship to survival.

## Methods

All data were downloaded from publicly available survey datasets on the Demographic and Health Surveys Program website (https://www.dhsprogram.com/). All survey methods were approved by the ICF Institutional Review Board (IRB) as well as equivalent boards within host countries and all information was gathered in accordance with the guidelines and regulations of these institutions. Further details are available at https://dhsprogram.com/methodology/Protecting-the-Privacy-of-DHS-Survey-Respondents.cfm.

Information about health services comes from the Service Provision Assessments (SPA). SPA are carried out in a sample of public and private [both not-for-profit, and for-profit] health facilities and are representative at the sub-national [regional/provincial] level. SPA includes information about infrastructure, facility staffing, staff training, types of services provided, commodities and fees.

We obtained data about births and survival from Demographic and Health Surveys (DHS) conducted in all the countries for which SPA data were available. DHS collect information about household demographics, health metrics, and child survival in a sample of households using a two-stage random sampling strategy. All mothers in sampled households are asked to give complete birth histories which document the date of birth and survival status for every child born to them.

Since SPA and DHS surveys are not carried out concurrently, we linked each birth from the DHS surveys with the health facility data in the SPA surveys. A birth was assigned to the respective SPA data for that country-region if there was an SPA survey available within 5 years of the recorded birth year, or to the nearest SPA if more than one was available. Births not linked to SPA data were excluded, all other births were included. All hospitals, health centers, and clinics from the SPA sample were included; dispensaries and stand-alone VCTs were excluded. A complete list of countries and years for DHS and SPA data is presented in Table S1A and the number of births per region-survey is presented in Table S1B.

Note that we are not linking births to a specific SPA facility, since the DHS and SPA surveys do not provide location data on a more granular level than the country-region. Instead, we are looking at the relationship between region-level mortality and health system characteristics, under the assumption that aa SPA survey conducted within a 5 years of the recorded birth year is representative of the relevant health context in which the child was born.

We defined two outcomes related to child survival—(1) survival past 12 months for every child born alive and (2) survival beyond 59 months for every child who survived to their first birthday. These correspond to standard definitions of infant mortality and child mortality.

We characterized health services based on three ‘domains’—geographic distribution, human resources as a measure of capacity to deliver quality, and out-of-pocket cost. We approximated the geographic distribution of health facilities within a country as the ratio of the proportion of that country’s total number of facilities located within that subnational region to the proportion of that country’s total population within that region. That is, a ratio of how concentrated that country’s health facilities are within each subnational region to how concentrated that country’s population is within that region. This is a measure of the degree to which the distribution of health facilities within a country aligns with the distribution of the population. Ratios > 1 then correspond to regions with greater per-capita concentration of health facilities. In addition, we included as a covariate the proportion of facilities with a region that were privately owned, as a proxy for the accessibility of private healthcare. We examine private health facilities specifically since their distribution is shaped by market forces (demand, ability to pay) rather than government policy.

The capacity to deliver quality services was represented by the proportion of facilities with a physician on staff and the proportion of clinical staff that had received training in the Integrated Management of Childhood Illnesses (IMCI). Healthcare costs to the patient were measured by the proportion of facilities in a region charging fees for three basic types of service—sick child visits, routine immunization, and normal delivery. We used standard sampling weights provided with each SPA dataset to account for survey design when calculating aggregated characteristics within a subnational region^21^.

The objective of this study was to estimate the relationship between infant and child survival and health systems factors. We identified control variables that may confound this relationship but do not lie on the causal pathway between health service delivery and the outcome of survival. As an example, we do not control for immunization status in our model even though it is related to survival because immunization coverage is likely related to the health services context. Similarly, we do not include measures of availability of specific interventions within each facility. Our goal is to estimate the association between the health services environment and survival rather than the impact of individual interventions. We examined correlations between our explanatory variables and ensured none were highly co-linear.

Separate log-normal accelerated failure time survival models were fit to each mortality outcome, with a normally distributed frailty term to account for the correlation of births with a common mother. The general log-linear representation of this model is given by:

$$log{T}_{ij}=\mu +{\beta }_{1}{x}_{1}+...+{\beta }_{p}{x}_{p}+{\alpha }_{j}+{\sigma \varepsilon }_{ij}$$

where ${T}_{ij}$ denotes the survival time of child *i* of mother *j*, $\mu$ denotes the intercept, ${\alpha }_{j}$ denotes the frailty term, and ${\sigma \varepsilon }_{ij}$ denotes the residuals, which follow a standard normal distribution and are multiplied by an arbitrary scaling factor $\sigma .$ The set of control and exposure variables included in the model are given by ${x}_{1}\dots {x}_{p}$ with coefficients ${\beta }_{1}\dots {\beta }_{p}$. We adjusted for individual-level characteristics that have been implicated in child survival: birth order, sex, maternal factors (education, age, marital status), and household wealth. We include an indicator for urban/rural and birth year to control for secular trends in child survival. Exposure variables were mean-centered and scaled by their interquartile range. The complete list of variables in the model is included in Table S3.

For infant mortality, child’s survival time was calculated as the time, in months, from the date of birth to the date of death, the date of the DHS interview, or the date of the child’s first birthday, whichever came first. For child mortality, the same approach was used beginning from the child’s first birthday up through their fifth birthday. Individuals whose interview date came before the relevant birthday were treated as censored. We analyzed survival to the first birthday and fifth birthday separately because we expected factors related to survival to differ between infants and young children. As a sensitivity analysis, we also fit a model for all deaths from date of birth to the child’s fifth birthday to assess the degree to which child mortality results may have been driven by conditioning on having survived to their first birthday. We assume risk of death is uniform within any given month^22^.

Without loss of generality, we re-parameterized the model such that the resulting coefficients are analogous to standard hazard ratios (i.e. ${\beta }_{i}=-{\gamma }_{i}$, where $\gamma$ denotes the standard coefficient of the accelerated failure time model). The parameter estimate for a given covariate is then interpreted as the multiplicative degree to which that covariate *decreases* the expected survival time of a child; so estimates greater than one correspond to a decrease of expected survival time and less than one correspond to an increase of expected survival time. Since this multiplicative factor is proportional to the true log-hazard, we interpret the exponentiated coefficients as approximates to the hazard ratio.

We elected not to use sampling weights. The individual-level sampling weights within DHS survey are normalized to sum to the sample size of that survey; thus, using the given weights would implicitly bias the results towards those surveys with larger sample sizes. Renormalizing the weights across the included surveys would require assumptions about the relationship of the probability of sampling both between and within countries over time. Since we did not feel that any such assumptions could be justified or verified, we took the pragmatic approach of using fixed-effect terms on country and birth year to control for systematic differences between countries and over time.

Finally, we note that we used Tanzania as the reference level for the country fixed effect in our models. We chose Tanzania since the proportion of childhood deaths in the country was approximately equal to the average across countries (see the last two rows of Table S2). This allows for intuitive comparisons of the fixed effect estimates for each country (see Table S3). The interpretation of the covariates of interest (i.e. SPA exposures) is not impacted by the choice of reference level.

All analyses were done in R, version 4.0.2, and models fit using the package ‘survival’, version 3.2–7 ^23^].

### Ethics approval and consent to participate

The DHS Program obtained ethical approvals for data collection and we obtained permission from the DHS Program to use these data. Duke Institutional Review Board approved this analysis as exempt from human subjects review.

## Results

We assembled a large, multi-national retrospective cohort of 256,031 children from seven African countries between 1995–2016. There were 13,629 deaths among infants before their first birthday and among children 12–59 months, there were an additional 5149 deaths.

Median maternal age at delivery was 26 years and most mothers had not completed primary school (Table 1, 59.1%). A quarter of households were located in urban areas. Mother characteristics differed slightly by country (Table S1)—there were fewer young mothers in Rwanda and more mothers completed secondary school in Namibia and Kenya. In Namibia, far fewer mothers were married and more households were located in urban areas.

**Table 1:** Child, mother and household characteristics of the retrospective cohort.

		*n* (%)
**Child characteristics**		
	Male	129,446 (50.6)
Birth order	1	59,114 (23.1)
	2–4	123,704 (48.3)
	> 4	73,213 (28.5)
Birth year	1995–1999	35,812 (14.0)
	2000–2003	52,622 (20.6)
	2004–2007	73,251 (28.6)
	2008–2011	84,844 (33.1)
	2012–2016	25,253 (9.9)
**Mother characteristics**		
Mother’s age	< 20	32,682 (12.8)
	20–24	74,429 (29.1)
	25–29	64,944 (25.4)
	30–34	46,783 (18.3)
	> 34	37,193 (14.5)
Mother’s education	None or some primary	151,247 (59.1)
	Finished primary	84,188 (32.9)
	Finished secondary or higher	20,596 (8.0)
Mother’s marital status	Married	180,975 (70.7)
**Household characteristics**		
Wealth quintile	Poorest	68,822 (26.9)
	Poorer	54,248 (21.2)
	Middle	49,211 (19.2)
	Richer	44,555 (17.4)
	Richest	39,195 (15.3)
Rural or urban	Urban	64,275 (25.1)
**Country**		
	Kenya	98,532 (38.5)
	Ghana	9474 (3.7)
	Namibia	10,604 (4.1)
	Rwanda	53,889 (20.0)
	Senegal	23,495 (9.2)
	Tanzania	41,823 (16.3)
	Uganda	18,215 (7.1)
Total		256,031

**Figure 1:**
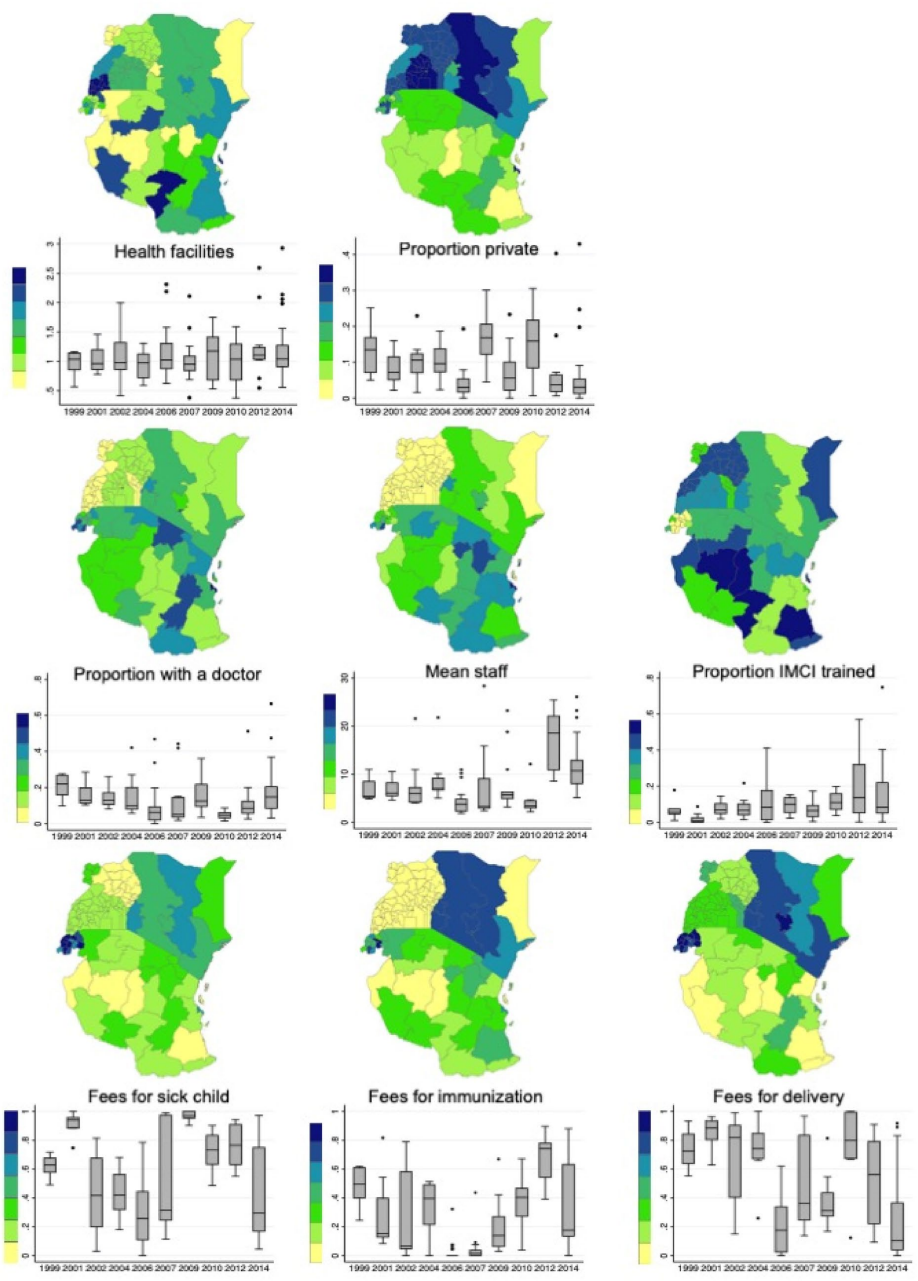
Health system variables by region and year. Maps of region-level means are shown for east Africa to demonstrate variability in space. The box plots show the median and range of each variable by year for all the countries contributing data in that year. Between 7 and 40 (mean = 15) regions are represented in each year of the box plots. Maps were generated in ArcMap 10.7 (Esri Inc., 2019, https://desktop.arcgis.com/en/arcmap/).

1–3 SPAs were conducted in each of the countries giving 150 region-years of paired SPA and survival information over the study period (Table 2). The ratio of facilities to total population within a region ranged from 0.97 to 1.19 (Fig. 1), and the average across region-years was highest for Senegal and lowest for Kenya. Overall, the proportion of private facilities was low, country-level means ranging from 0.04 in Tanzania to 0.14 in Uganda. Few facilities had doctors; 9 per 100 in Uganda, up to 17 per 100 in Rwanda. Senegal had the highest levels of IMCI training at 20% on average trained per facility. Namibia had the highest proportion of facilities charging fees for sick child visits (97%), while Rwanda had the highest proportion charging fees for delivery (86%). Fees for immunization were least common, although 69% of facilities in Senegal did charge for routine immunization. The fewest facilities charged fees (for any service) in Tanzania and Uganda.

**Table 2:** Country-level health systems characteristics.

Country	# Region-years	Access		Quality		Cost		
		Ratio of facilities to population	Proportion of private facilities	Proportion of facilities with doctors	Mean proportion with IMCI training	Proportion charging sick child fees	Proportion charging immunization fees	Proportion charging delivery fees
Ghana	10	1.11 (0.46)	0.10 (0.06)	0.14 (0.06)	0.08 (0.04)	0.43 (0.28)	0.24 (0.30)	0.69 (0.29)
Kenya	23	0.97 (0.29)	0.13 (0.07)	0.13 (0.10)	0.09 (0.06)	0.59 (0.18)	0.39 (0.18)	0.74 (0.22)
Namibia	13	1.15 (0.43)	0.08 (0.07)	0.16 (0.09)	0.07 (0.05)	0.97 (0.03)	0.2 (0.18)	0.37 (0.17)
Rwanda	17	1.04 (0.23)	0.12 (0.08)	0.17 (0.09)	0.03 (0.04)	0.93 (0.08)	0.23 (0.22)	0.86 (0.09)
Senegal	28	1.19 (0.46)	0.07 (0.11)	0.12 (0.11)	0.2 (0.18)	0.78 (0.14)	0.69 (0.14)	0.51 (0.28)
Tanzania	52	1.14 (0.45)	0.04 (0.04)	0.15 (0.15)	0.12 (0.12)	0.26 (0.17)	0.14 (0.09)	0.16 (0.16)
Uganda	7	1.06 (0.55)	0.14 (0.05)	0.09 (0.15)	0.11 (0.04)	0.28 (0.14)	0.01 (0.03)	0.31 (0.16)

Mean (SD) The ratio of facilities to population serves as a proxy measure for physical access and describe the concentration of facilities relative to the concentration of population in that region. The proportion of private facilities describes the relative availability of private healthcare. The proportion of facilities with a doctor, the mean number of staff, and the proportion of staff per facility trained in Integrated management of childhood illnesses (IMCI) represent the capacity to deliver quality services. Finally, the proportion of facilities charging fees for acute sick child visits, routine immunization (scheduled prevention visits) and normal delivery represent the out-of-pocket cost of basic.

**Figure 2:**
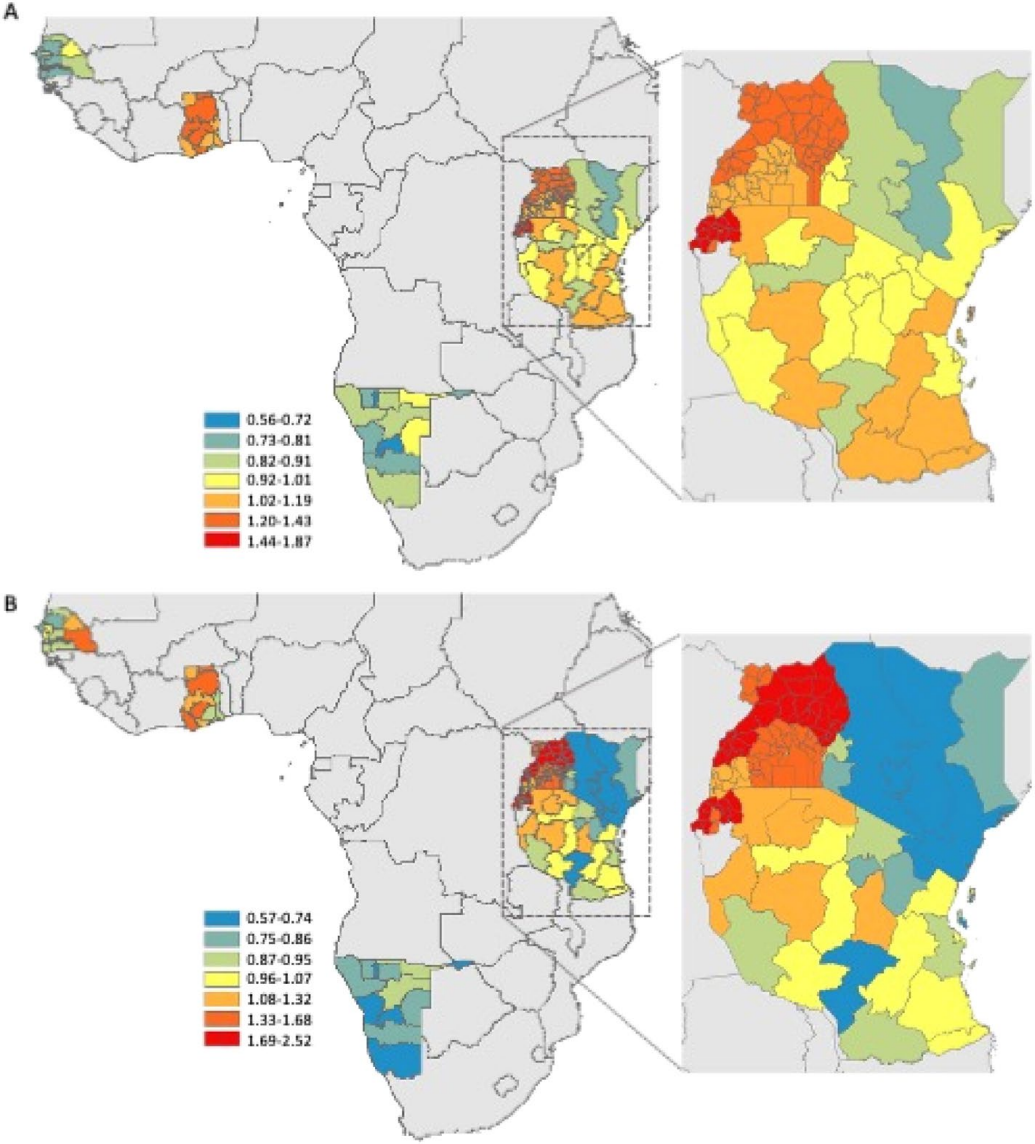
Map of study area with estimated hazard ratio for (**A**) child and (**B**) infant mortality per region averaged over the study period relative to the median value. East Africa is enlarged (inset) to better appreciate the inter and intra-country differences in risk of death. Maps were generated in ArcMap 10.7 (Esri Inc., 2019, https://desktop.arcgis.com/en/arcmap/).

**Figure 3:**
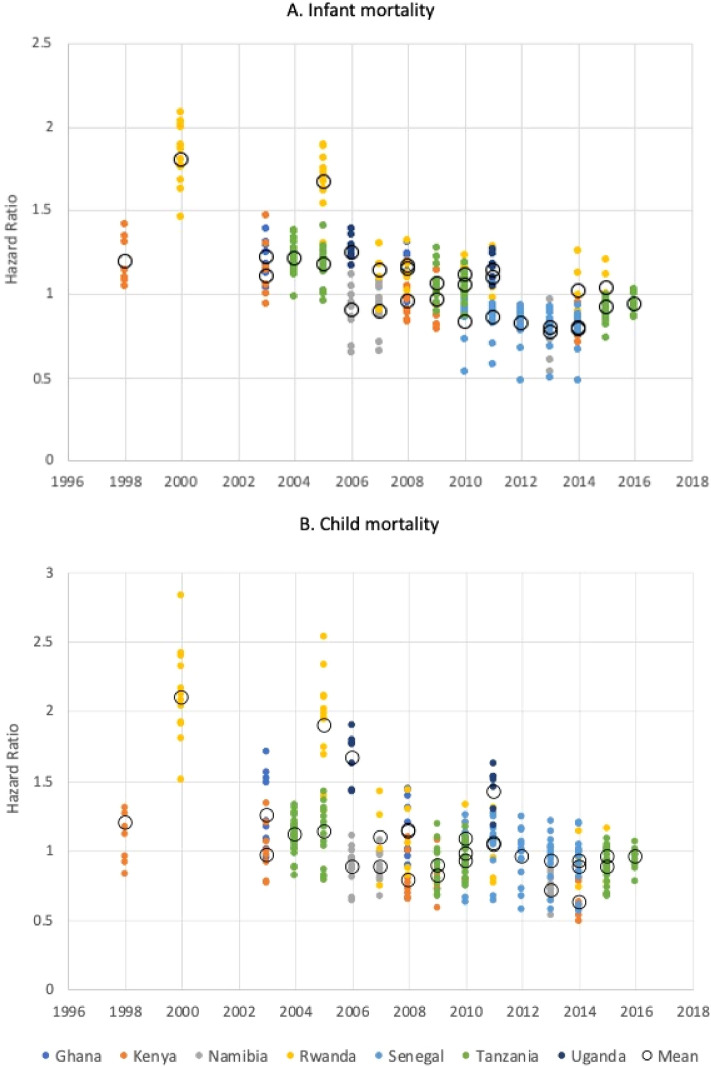
(**A**) Estimated hazard ratio for risk of death (**A**) before 12 months of age and (**B**) between 12 and 60 months of age over time by country (relative to the median of all region-years).

Infant and child survival differed between countries and between regions within countries (Fig. 2). Overall, the risk of childhood death declined over time (Fig. 3). Fully-adjusted models showed country-level deceleration factors ranging from 0.82 in Kenya up to 1.88 in Uganda; this indicates that average child survival time was 18% longer in Kenya and 88% shorter in Uganda relative to the reference country (Tanzania). At the individual-level, increasing maternal education, being married, and increasing household wealth were protective (Supplemental Table 3).

**Table 3:** Unadjusted and adjusted estimated hazard ratios (95% Confidence interval) for the effect of health system characteristics on time to death for infants and children.

	Child survival		Infant survival	
Covariate	Unadjusted	Adjusted^1^	Unadjusted	Adjusted^1^
**Region level health systems factors**				
**Access**				
Ratio facilities to population^2^	0.97 (0.94, 0.99)	0.97 (0.94, 0.99)	1.01 (0.99, 1.04)	1.02 (0.99, 1.04)
Proportion private facilities	0.78 (0.75, 0.8)	0.81 (0.79, 0.84)	0.83 (0.81, 0.86)	0.87 (0.85, 0.9)
**Quality**				
Proportion with a doctor	1.1 (1.07, 1.12)	1.11 (1.08, 1.14)	1.05 (1.03, 1.07)	1.03 (1.01, 1.05)
Proportion trained in IMCI^3^	0.95 (0.92, 0.98)	0.99 (0.96, 1.02)	0.96 (0.94, 0.99)	1.01 (0.98, 1.03)
**Cost**				
Proportion charging fees for sick child services^4^	0.82 (0.74, 0.91)	0.98 (0.88, 1.1)	0.82 (0.75, 0.91)	1.03 (0.94, 1.14)
Proportion charging fees for immunization^4^	1.33 (1.25, 1.42)	1.20 (1.12, 1.28)	1.05 (0.99, 1.11)	0.96 (0.9, 1.01)
Proportion charging fees for delivery^4^	0.8 (0.72, 0.89)	0.82 (0.74, 0.91)	1.12 (1.03, 1.23)	1.11 (1.01, 1.21)

These estimates are exponentiated coefficients from accelerated failure time models and approximate the hazard ratio. Full model results including all covariates is presented in Supplemental Table 3.

^1^Adjusted for child-level (male, birth order and birth year), maternal-level (maternal age, education and marital status) and household-level (urban, wealth score) factors.

^2^The ratio of facilities in a region divided by the ratio of the population in the same region.

^3^Proportion of health staff trained in Integrated Management of Childhood Illness per facility averaged across all facilities in the region.

^4^Proportion of facilities in a region charging any fees for these types of services, including for consultation, registration, health card, drugs, vaccines, syringes or needles.

Specific aspects of the health services context were related to survival and these patterns were distinct for infant and child mortality (Table 3). In fully-adjusted survival models, higher ratio of health facilities relative to the population was associated with improved child survival (0.97, 95% CI 0.94–0.99), but not infant survival. The proportion of private facilities in a region was associated with improved survival for both children (0.81, 95% CI 0.79–0.84) and infants (0.87, 95% CI 0.85–0.9). However, health workforce indicators were not associated with improved survival. In particular, the proportion of facilities with a doctor was negatively correlated with both child (1.11, 95% CI 1.08–1.14) and infant (1.03, 95% CI 1.01–1.05) survival.

Survival was also related to the cost of care. Moving from the first to the third quartile of prevalence of delivery fees (10–77%) was associated with an 11% (95% CI 1–21%) increase in risk of death in infancy in the fully adjusted model. A similar scaled increase in the proportion of facilities charging immunization fees (from 27.5 to 73.1% of facilities charging fees) was associated with 20% (95% CI 12–28%) increased risk of death among children. Conversely, delivery fees were associated with improved child survival (0.82, 95% CI 0.74–0.91).

## Discussion

As the global community solidifies its commitment to UHC, vital initiatives to extend coverage^24^ will likely be crippled by insufficient health systems and there is an urgent need for evidence to guide investment in health systems themselves^25,26^. In order to help identify critical shortcomings that may directly impact child survival, we analyzed data from > 250,000 children born over 20 years in seven countries and show that infant and child survival are correlated with specific aspects of health systems context. In our analysis, physical distribution of health services is an important determinant of child survival, a finding which agrees with other studies using similar data sources^27^ and with results which estimate that, in sub-Saharan Africa, a higher proportion of avertable deaths is associated with poor access than poor quality^28^. The association between poor access and mortality could reflect limited physical access in terms of distance or travel time or poor distribution of facilities resulting in crowding and unmanageable patient volumes. The positive effect of private facilities on survival, even after adjusting for wealth and urban context, may be attributable to documented higher quality in private facilities in some countries^13,29^ or potentially a reflection of the private sector responding to a demand for healthcare that the public sector has not met. Although the proportion of facilities that were private was generally low, the effect size was important, further highlighting the value of engaging the private sector in UHC strategies.

The lack of association of child survival with curative service fees coupled with an association with health infrastructure together suggest that expansion of insurance programs may be insufficient to substantially reduce mortality in this vulnerable group. In contrast, the negative association of delivery fees with infant survival points to a key opportunity to reduce neonatal and infant mortality by improving the affordability of safe delivery^30^. The positive association between delivery fees and child survival may be a survivorship effect whereby higher mortality during infancy in areas where delivery fees are prevalent may lead to lower apparent mortality after one year of age. This interpretation is supported by the lack of a positive association between survival and delivery fees in our sensitivity analysis of survival from birth to the fifth birthday [i.e. the effect is only observed when conditioning on survival to one year]. The negative association between child survival and immunization fees may reflect low uptake of immunization when out-of-pocket payments are required and points to the importance of providing these high-impact, preventive services free of charge. We previously showed that fees for sick child visits reduced child survival in Kenya, but this effect is not seen in the current multi-country analysis^31^.

Doctors and IMCI-trained staff were not associated with improved survival. A comparison of child mortality in Mozambique also demonstrated a protective effect of higher density of nurses and midwives, but not physicians^32^. On the surface, the negative association between survival and prevalence of a doctor seems to contradict inter-country comparisons of density of doctors on child mortality^33,34^. However, when examined more closely, the protective effect of physician density is only observed in high-functioning health systems of low-mortality, high-income countries^35^. Our finding is consistent with observations by others that the presence of a doctor has no effect on the quality of acute child care, antenatal care, or delivery services in under-resourced settings^36^. We did not observe a strong correlation between doctors and private facilities or fees which, if present, could confound this result (Table S4), however it is possible that proportion of doctors indicates higher concentrations of larger facilities such as hospitals relative to clinics. Lack of an association between IMCI training and survival agrees with reports that training may have limited impact on quality of care^37,38^ as well as evaluations of IMCI training itself ^37^. These results have important implications—first, many life-saving interventions can be effectively deployed by nurses and clinical officers and higher-level formal training is not required to improve child survival. Second, improving the skill level of health facility staff may not have the expected impact without the tools and motivation to put the training into practice.

Results should be interpreted while considering study limitations. Data for this study came from a subset of sub-Saharan African countries, based on the availability of SPA, potentially limiting the generalizability. Health insurance programs may affect the relationship between fees and mortality via prepayment or premiums. Ghana has a comparatively high rate of enrollment in national health insurance and Rwanda has high enrollment in community-based health insurance. Geographic distribution of health facilities relative to population is an imperfect measure of physical access. We may not be able to distinguish between regions of the same population and number of facilities but very different geographic extent which would lead to potentially large unmeasured differences in physical access. Mortality is assessed through birth histories that are subject to recall bias and survivorship bias.

Finally, our exposure variables are aggregated at the sub-national level and we cannot relate each child to their nearest health facility. This restricts us from leveraging more fine-scaled heterogeneities and assigns each birth to the region-level mean, likely biasing effect estimates towards the null. In addition, we did not account for the location of birth at the individual or region level which could be influenced by factors other than health system characteristics, including cultural practices and norms. Despite this imprecision, we are still able to measure substantial correlation with survival outcomes.

Our analysis has several important strengths. First, we move beyond evaluation of quality or readiness and relate health systems characteristics to child survival outcomes. Second, we examine this relationship at the sub-national level over two decades across seven countries, incorporating data from multiple surveys within the same country. This allows us to leverage documented high levels of heterogeneity in child survival between subnational regions^20^ to quantify the impact of health systems context on survival outcomes. Longitudinal analysis ensures that we capture the effect of change in health systems on mortality, for example the expansion of ARTs and HIV care, investment in infrastructure during the MDG era, the evolution of health insurance, and the abolition of user fees in several countries. These factors enhance the generalizability of our results.

UHC is emerging as a key pillar for achieving Sustainable Development Goal 3 and ending preventable childhood deaths. Ninety-percent of the Disease Control Priorities-3 (DCP3) essential UHC interventions are delivered through primary health care facilities^11^. As a result, the impact of UHC will depend heavily on the characteristics of the underlying health system and it is important to understand the relationship between health systems factors and health outcomes. Here, we isolate the impact of health systems characteristics and directly measure the impact on population-based survival outcomes. Our findings suggest key areas for further research with respect to realizing the potential of UHC, including (1) the role of public subsidies or health insurance to mitigate the negative impact of user fees for immunization and delivery, (2) investment in physical infrastructure to improve access, (3) engaging the private sector to achieve UHC goals, and (4) closing the human resource gap with basic cadres of health care workers.

## Supplementary Information

Supplementary Table S1A.

Supplementary Table S1B.

Supplementary Table S2.

Supplementary Table S3.

Supplementary Table S4.

Supplementary Table S5.
