# Supplementary material for: Effect of health systems context on infant and child mortality in sub-Saharan Africa from 1995 to 2015, a longitudinal cohort analysis
Source: Sci Rep. 2021 Aug 11;11:16263. doi: 10.1038/s41598-021-95886-8 (PMC8357794; doi:10.1038/s41598-021-95886-8)
Supplement: Supplementary file 1 — Supplementary Table S1A. [file 41598_2021_95886_MOESM1_ESM.docx]

| Table S1A: Data sources for both birth history (DHS data) and health systems (SPA) by country and year | | | | | | | | | | | | | | | | | | | |  |
| --- | --- | --- | --- | --- | --- | --- | --- | --- | --- | --- | --- | --- | --- | --- | --- | --- | --- | --- | --- | --- |
|  |  | 1998 | 1999 | 2000 | 2001 | 2002 | 2003 | 2004 | 2005 | 2006 | 2007 | 2008 | 2009 | 2010 | 2011 | 2012 | 2013 | 2014 | 2015 | 2016 |
| KENYA | DHS |  |  |  |  |  |  |  |  |  |  |  |  |  |  |  |  |  |  |  |
|  | SPA | |  |  |  |  |  |  |  |  |  |  |  |  |  |  |  |  |  |  |
| NAMIBIA | DHS | |  |  |  |  |  |  |  |  |  |  |  |  |  |  |  |  |  |  |
|  | SPA | |  |  |  |  |  |  |  |  |  |  |  |  |  |  |  |  |  |  |
| GHANA | DHS |  |  |  |  |  |  |  |  |  |  |  |  |  |  |  |  |  |  |  |
|  | SPA | |  |  |  |  |  |  |  |  |  |  |  |  |  |  |  |  |  |  |
| RWANDA | DHS | |  |  |  |  |  |  |  |  |  |  |  |  |  |  |  |  |  |  |
|  | SPA | |  |  |  |  |  |  |  |  |  |  |  |  |  |  |  |  |  |  |
| SENEGAL | DHS | |  |  |  |  |  |  |  |  |  |  |  |  |  | * |  | * |  |  |
|  | SPA | |  |  |  |  |  |  |  |  |  |  |  |  |  |  |  |  |  |  |
| TANZANIA | DHS | |  |  |  |  |  |  |  |  |  |  |  |  |  |  |  |  |  |  |
|  | SPA | |  |  |  |  |  |  |  |  |  |  |  |  |  |  |  |  |  |  |
| UGANDA | DHS | |  |  |  |  |  |  |  |  |  |  |  |  |  |  |  |  |  |  |
|  | SPA | |  |  |  |  |  |  |  |  |  |  |  |  |  |  |  |  |  |  |
|  |  |  |  |  |  |  |  |  |  |  |  |  |  |  |  |  |  |  |  |  |
|  | *=rolling DHS | | |  |  |  |  |  |  |  |  |  |  |  |  |  |  |  |  |  |

Notes:

Kenya 1999: Northeastern province not included in survey

Senegal 2012-13: The 2012-13 and 2014 Senegal SPAs represent years 1 and 2 of a 5-year pilot continuous SPA

Tanzania 2014: In 2012, Tanzania created four new regions (Njombe, Katavi, Simiyu, and Geita) from portions of previously existing regions. When linking the 2014 SPA to the 2010 DHS, we linked Njombe to Iringa, Katavi to Rukwa, and Simiyu to Shinyanga. We were forced to exclude Geita since it could not be unambiguously linked to a single region in the 2010 DHS.

Uganda 2007: The 2007 Uganda SPA used geographic regions, not administrative ones, for sampling. Not all regions could be linked to regions in the 2000, 2006, or 2011 DHS reports. In addition, the 2006 DHS oversampled from two special areas of interest: Karamoja and internally displaced persons (IDP) camps.
