# Supplementary material for: Effect of health systems context on infant and child mortality in sub-Saharan Africa from 1995 to 2015, a longitudinal cohort analysis
Source: Sci Rep. 2021 Aug 11;11:16263. doi: 10.1038/s41598-021-95886-8 (PMC8357794; doi:10.1038/s41598-021-95886-8)
Supplement: Supplementary file 2 — Supplementary Table S1B. [file 41598_2021_95886_MOESM2_ESM.docx]

| Table S1B: Sample sizes (number of births linked to an SPA) per region-survey. Note that for surveys straddling multiple years (e.g. Kenya DHS 2008-2009) we do not distinguish by survey-year, since it is not used in our analysis. | | | | | | | | | | | | | | | | | | | | |
| --- | --- | --- | --- | --- | --- | --- | --- | --- | --- | --- | --- | --- | --- | --- | --- | --- | --- | --- | --- | --- |
| Country | Region | 1998 | 1999 | 2000 | 2001 | 2002 | 2003 | 2004 | 2005 | 2006 | 2007 | 2008 | 2009 | 2010 | 2011 | 2012 | 2013 | 2014 | 2015 | 2016 |
| Kenya | Central | 323 |  |  |  |  | 1189 |  |  |  |  | 1322  2150  1996  929  1877  2722  1995  1490 | |  |  |  |  | 5315 |  |  |
|  | Coast | 587 |  |  |  |  | 1151 |  |  |  |  |  |  |  |  |  |  | 8954 |  |  |
|  | Eastern | 526 |  |  |  |  | 1140 |  |  |  |  |  |  |  |  |  |  | 11122 |  |  |
|  | Nairobi | 136 |  |  |  |  | 867 |  |  |  |  |  |  |  |  |  |  | 1423 |  |  |
|  | Northeastern | n/a |  |  |  |  | 576 |  |  |  |  |  |  |  |  |  |  | 4712 |  |  |
|  | Nyanza | 678 |  |  |  |  | 1911 |  |  |  |  |  |  |  |  |  |  | 10719 |  |  |
|  | Rift Valley | 1091 |  |  |  |  | 1287 |  |  |  |  |  |  |  |  |  |  | 22108 |  |  |
|  | Western | 534 |  |  |  |  | 818 |  |  |  |  |  |  |  |  |  |  | 6884 |  |  |
| Namibia | Caprivi |  |  |  |  |  |  |  |  | 153  111  112  98  336  220  115  235  147  195  184  203  196 | |  |  |  |  |  | 644 |  |  |  |
|  | Erongo |  |  |  |  |  |  |  |  |  |  |  |  |  |  |  | 631 |  |  |  |
|  | Hardap |  |  |  |  |  |  |  |  |  |  |  |  |  |  |  | 516 |  |  |  |
|  | Karas |  |  |  |  |  |  |  |  |  |  |  |  |  |  |  | 658 |  |  |  |
|  | Kavango |  |  |  |  |  |  |  |  |  |  |  |  |  |  |  | 860 |  |  |  |
|  | Khomas |  |  |  |  |  |  |  |  |  |  |  |  |  |  |  | 639 |  |  |  |
|  | Kunene |  |  |  |  |  |  |  |  |  |  |  |  |  |  |  | 735 |  |  |  |
|  | Ohangewena |  |  |  |  |  |  |  |  |  |  |  |  |  |  |  | 747 |  |  |  |
|  | Omakeke |  |  |  |  |  |  |  |  |  |  |  |  |  |  |  | 589 |  |  |  |
|  | Omusati |  |  |  |  |  |  |  |  |  |  |  |  |  |  |  | 589 |  |  |  |
|  | Oshana |  |  |  |  |  |  |  |  |  |  |  |  |  |  |  | 455 |  |  |  |
|  | Oshitoto |  |  |  |  |  |  |  |  |  |  |  |  |  |  |  | 568 |  |  |  |
|  | Otjozondjupa |  |  |  |  |  |  |  |  |  |  |  |  |  |  |  | 667 |  |  |  |
| Ghana | Ashanti |  |  |  |  |  | 662 |  |  |  |  | 765 |  |  |  |  |  |  |  |  |
|  | Brong Ahafo |  |  |  |  |  | 526 |  |  |  |  | 452 |  |  |  |  |  |  |  |  |
|  | Central |  |  |  |  |  | 280 |  |  |  |  | 370 |  |  |  |  |  |  |  |  |
|  | Eastern |  |  |  |  |  | 368 |  |  |  |  | 440 |  |  |  |  |  |  |  |  |
|  | Greater Accra |  |  |  |  |  | 372 |  |  |  |  | 452 |  |  |  |  |  |  |  |  |
|  | Northern |  |  |  |  |  | 700 |  |  |  |  | 761 |  |  |  |  |  |  |  |  |
|  | Upper East |  |  |  |  |  | 427 |  |  |  |  | 417 |  |  |  |  |  |  |  |  |
|  | Upper West |  |  |  |  |  | 315 |  |  |  |  | 514 |  |  |  |  |  |  |  |  |
|  | Volta |  |  |  |  |  | 300 |  |  |  |  | 467 |  |  |  |  |  |  |  |  |
|  | Western |  |  |  |  |  | 394 |  |  |  |  | 492 |  |  |  |  |  |  |  |  |
| Rwanda 1 | Butare |  |  | 450 |  |  |  |  | 943 |  |  |  |  |  |  |  |  |  |  |  |
|  | Byumba |  |  | 500 |  |  |  |  | 1101 |  |  |  |  |  |  |  |  |  |  |  |
|  | Cyangugu |  |  | 501 |  |  |  |  | 1008 |  |  |  |  |  |  |  |  |  |  |  |
|  | Gikongoro |  |  | 400 |  |  |  |  | 1097 |  |  |  |  |  |  |  |  |  |  |  |
|  | Gisenyi |  |  | 426 |  |  |  |  | 1181 |  |  |  |  |  |  |  |  |  |  |  |
|  | Gitarama |  |  | 454 |  |  |  |  | 1259 |  |  |  |  |  |  |  |  |  |  |  |
|  | Kibungo |  |  | 465 |  |  |  |  | 1105 |  |  |  |  |  |  |  |  |  |  |  |
|  | Kibuye |  |  | 467 |  |  |  |  | 1315 |  |  |  |  |  |  |  |  |  |  |  |
|  | Kigali Rurale |  |  | 540 |  |  |  |  | 1324 |  |  |  |  |  |  |  |  |  |  |  |
|  | Kigali Ville |  |  | 737 |  |  |  |  | 1332 |  |  |  |  |  |  |  |  |  |  |  |
|  | Ruhengeri |  |  | 606 |  |  |  |  | 1296 |  |  |  |  |  |  |  |  |  |  |  |
|  | Umutara |  |  | 326 |  |  |  |  | 1140 |  |  |  |  |  |  |  |  |  |  |  |
| Rwanda 2 | Eastern |  |  |  |  |  |  |  |  |  | 1524  1374  581  1358  865 | |  | 3442  3624  1532  3601  2259 | |  |  | 3321  1512  2101  3537  3285 | |  |
|  | Kigali City |  |  |  |  |  |  |  |  |  |  |  |  |  |  |  |  |  |  |  |
|  | Northern |  |  |  |  |  |  |  |  |  |  |  |  |  |  |  |  |  |  |  |
|  | Southern |  |  |  |  |  |  |  |  |  |  |  |  |  |  |  |  |  |  |  |
|  | Western |  |  |  |  |  |  |  |  |  |  |  |  |  |  |  |  |  |  |  |
| Senegal | Dakar |  |  |  |  |  |  |  |  |  |  |  |  | 613  604  113  238  311  171  262  725  720  725  184  302  213  80 | | | 584  1119  956  1195  1331  668  1031  946  919  803  817  1046  1196  525 | |  |  |
|  | Diourbel |  |  |  |  |  |  |  |  |  |  |  |  |  |  |  |  |  |  |  |
|  | Fatick |  |  |  |  |  |  |  |  |  |  |  |  |  |  |  |  |  |  |  |
|  | Kaffrine |  |  |  |  |  |  |  |  |  |  |  |  |  |  |  |  |  |  |  |
|  | Kaolack |  |  |  |  |  |  |  |  |  |  |  |  |  |  |  |  |  |  |  |
|  | Kedougou |  |  |  |  |  |  |  |  |  |  |  |  |  |  |  |  |  |  |  |
|  | Kolda |  |  |  |  |  |  |  |  |  |  |  |  |  |  |  |  |  |  |  |
|  | Louga |  |  |  |  |  |  |  |  |  |  |  |  |  |  |  |  |  |  |  |
|  | Matam |  |  |  |  |  |  |  |  |  |  |  |  |  |  |  |  |  |  |  |
|  | Saint-Louis |  |  |  |  |  |  |  |  |  |  |  |  |  |  |  |  |  |  |  |
|  | Sedhiou |  |  |  |  |  |  |  |  |  |  |  |  |  |  |  |  |  |  |  |
|  | Tambacounda |  |  |  |  |  |  |  |  |  |  |  |  |  |  |  |  |  |  |  |
|  | Thiès |  |  |  |  |  |  |  |  |  |  |  |  |  |  |  |  |  |  |  |
|  | Ziguinchor |  |  |  |  |  |  |  |  |  |  |  |  |  |  |  |  |  |  |  |
| Tanzania | Arusha |  |  |  |  |  |  | 186  105  191  140  259  237  202  251  110  221  150  124  217  260  230  193  136  152  247  158  252  181  329  234  303  160 | |  |  |  | 425  304  501  356  526  598  497  529  255  568  399  370  502  716  440  547  404  335  681  417  560  409  819  574  736  352 | |  |  |  |  | 714  917  700  1236  916  757  679  1026  481  750  582  587  920  1185  604  776  638  519  926  590  2147  676  2605  883  1250  711 | |
|  | Dar es Salaam |  |  |  |  |  |  |  |  |  |  |  |  |  |  |  |  |  |  |  |
|  | Dodoma |  |  |  |  |  |  |  |  |  |  |  |  |  |  |  |  |  |  |  |
|  | Iringa |  |  |  |  |  |  |  |  |  |  |  |  |  |  |  |  |  |  |  |
|  | Kagerka |  |  |  |  |  |  |  |  |  |  |  |  |  |  |  |  |  |  |  |
|  | Kaskazini Pemba |  |  |  |  |  |  |  |  |  |  |  |  |  |  |  |  |  |  |  |
|  | Kaskazini Unguja |  |  |  |  |  |  |  |  |  |  |  |  |  |  |  |  |  |  |  |
|  | Kigoma |  |  |  |  |  |  |  |  |  |  |  |  |  |  |  |  |  |  |  |
|  | Kilimanjaro |  |  |  |  |  |  |  |  |  |  |  |  |  |  |  |  |  |  |  |
|  | Kusini Pemba |  |  |  |  |  |  |  |  |  |  |  |  |  |  |  |  |  |  |  |
|  | Kusini Unguja |  |  |  |  |  |  |  |  |  |  |  |  |  |  |  |  |  |  |  |
|  | Lindi |  |  |  |  |  |  |  |  |  |  |  |  |  |  |  |  |  |  |  |
|  | Manyara |  |  |  |  |  |  |  |  |  |  |  |  |  |  |  |  |  |  |  |
|  | Mara |  |  |  |  |  |  |  |  |  |  |  |  |  |  |  |  |  |  |  |
|  | Mbeya |  |  |  |  |  |  |  |  |  |  |  |  |  |  |  |  |  |  |  |
|  | Mjini Magharib |  |  |  |  |  |  |  |  |  |  |  |  |  |  |  |  |  |  |  |
|  | Morogoro |  |  |  |  |  |  |  |  |  |  |  |  |  |  |  |  |  |  |  |
|  | Mtwara |  |  |  |  |  |  |  |  |  |  |  |  |  |  |  |  |  |  |  |
|  | Mwanza |  |  |  |  |  |  |  |  |  |  |  |  |  |  |  |  |  |  |  |
|  | Pwani |  |  |  |  |  |  |  |  |  |  |  |  |  |  |  |  |  |  |  |
|  | Rukwa |  |  |  |  |  |  |  |  |  |  |  |  |  |  |  |  |  |  |  |
|  | Ruvuma |  |  |  |  |  |  |  |  |  |  |  |  |  |  |  |  |  |  |  |
|  | Shinyanga |  |  |  |  |  |  |  |  |  |  |  |  |  |  |  |  |  |  |  |
|  | Singida |  |  |  |  |  |  |  |  |  |  |  |  |  |  |  |  |  |  |  |
|  | Tabora |  |  |  |  |  |  |  |  |  |  |  |  |  |  |  |  |  |  |  |
|  | Tanga |  |  |  |  |  |  |  |  |  |  |  |  |  |  |  |  |  |  |  |
| Uganda | Central |  |  |  |  |  |  |  |  | 1035 |  |  |  |  | 2407 |  |  |  |  |  |
|  | East Central |  |  |  |  |  |  |  |  | 733 |  |  |  |  | 1541 |  |  |  |  |  |
|  | Eastern/Northern |  |  |  |  |  |  |  |  | 2101 |  |  |  |  | 2959 |  |  |  |  |  |
|  | Kampala |  |  |  |  |  |  |  |  | 360 |  |  |  |  | 971 |  |  |  |  |  |
|  | Southwest |  |  |  |  |  |  |  |  | 616 |  |  |  |  | 1370 |  |  |  |  |  |
|  | West Nile |  |  |  |  |  |  |  |  | 524 |  |  |  |  | 1382 |  |  |  |  |  |
|  | Western |  |  |  |  |  |  |  |  | 685 |  |  |  |  | 1531 |  |  |  |  |  |
