# Supplementary material for: Effect of health systems context on infant and child mortality in sub-Saharan Africa from 1995 to 2015, a longitudinal cohort analysis
Source: Sci Rep. 2021 Aug 11;11:16263. doi: 10.1038/s41598-021-95886-8 (PMC8357794; doi:10.1038/s41598-021-95886-8)
Supplement: Supplementary file 3 — Supplementary Table S2. [file 41598_2021_95886_MOESM3_ESM.docx]

Table S2: Child, mother and household characteristics of the retrospective cohort by country.

|  | | TOTAL N(%) | KENYA N(%) | GHANA N(%) | NAMIBIA N(%) | RWANDA N(%) | SENEGAL N(%) | TANZANIA N(%) | UGANDA N(%) |
| --- | --- | --- | --- | --- | --- | --- | --- | --- | --- |
| **CHILD CHARACTERISTICS** | | | | | | | | | |
| Sex | Male | 129 446 (50.6) | 49 918 (50.7) | 4811 (50.8) | 5342 (50.4) | 27 417 (50.9) | 11 950 (50.9) | 20 954 (50.1) | 9,054 (49.7) |
| Birth order | 1 | 59 114 (23.1) | 24 599 (25.0) | 2169 (22.9) | 3323 (31.3) | 11 839 (22.0) | 4905 (20.9) | 8977 (21.5) | 3302 (18.1) |
|  | 2-4 | 123 704 (48.3) | 49 530 (50.3) | 4515 (47.7) | 5539 (52.2) | 25 644 (47.6) | 11 007 (46.8) | 19 662 (47.0) | 7807 (42.9) |
|  | 5+ | 73 213 (28.5) | 24 403 (25.7) | 2790 (29.4) | 1741 (16.4) | 16 407 (30.4) | 7583 (32.3) | 13 184 (31.5) | 7106 (39.0) |
| Birth year | 1995-1999 | 35 812 (14.0) | 24 128 (24.5) | 2482 (26.2) | 0 (0) | 9202 (17.1) | 0 (0) | 0 (0) | 0 (0) |
|  | 2000-2003 | 52 622 (20.6) | 22 841 (23.2) | 5257 (55.5) | 0 (0) | 12 484 (23.2) | 0 (0) | 9053 (21.6) | 2987 (16.4) |
|  | 2004-2007 | 73 251 (28.6) | 21 805 (22.1) | 1735 (18.3) | 5035 (47.5) | 20 548 (38.1) | 0 (0) | 14 277 (34.1) | 9851 (54.1) |
|  | 2008-2011 | 84 844 (33.1) | 18 892 (19 2) | 0 (0) | 3829 (36.1) | 11 655 (21.6) | 18 518 (78.8) | 10 822 (25.9) | 5377 (29.5) |
|  | 2012-2016 | 25 253 (9.9) | 10 866 (11.0) | 0 (0) | 1739 (16.4) | 0 (0) | 4977 (21.2) | 7671 (18.3) | 0 (0) |
| **MOTHER & HOUSEHOLD CHARACTERISTICS** | | | | | | | | | |
| Mother’s age | < 20 | 32 682 (12.8) | 16 390 (16.6) | 1004 (10.6) | 1472 (13.9) | 2727 (5.1) | 2827 (12.0) | 5628 (13.5) | 2634 (14.5) |
|  | 20-24 | 74 429 (29.1) | 31 324 (31.8) | 2323 (24.5) | 2985 (28.2) | 14 275 (26.5) | 6458 (27.5) | 11 662 (27.9) | 5402 (29.7) |
|  | 25-29 | 64 944 (25.4) | 24 800 (25.2) | 2398 (25.3) | 2473 (23.3) | 15 129 (28.1) | 5828 (24.8) | 9867 (23.5) | 4449 (24.4) |
|  | 30-34 | 46 783 (18.3) | 16 245 (16.5) | 1971 (20.8) | 1968 (18.6) | 11 119 (20.6) | 4347 (18.5) | 7942 (19.0) | 3191 (17.5) |
|  | >34 | 37 193 (14.5) | 9773 (9.9) | 1778 (18.8) | 1705 (16.1) | 10 639 (19.7) | 4035 (17.2) | 6724 (16.1) | 2539 (13.9) |
| Mother’s education | None or some primary | 151 247 (59.1) | 52 193 (53.0) | 5808 (61.3) | 3088 (29.1) | 35 592 (73.5) | 20 999 (89.4) | 16 707 (40.0) | 12 860 (70.6) |
|  | Finished primary | 84 188 (32.9) | 32 474 (32.9) | 3255 (34.4) | 5678 (53.6) | 12 624 (23.4) | 2301 (9.8) | 23 227 (55.5) | 4629 (25.4) |
|  | Finished secondary or higher | 20 596 (8.0) | 13.865 (14.1) | 411 (4.3) | 1837 (17.3) | 1673 (3.1) | 195 (0.8) | 1889 (4.5) | 726 (4.0) |
| Marital Status | Married | 180 975 (70.7) | 77 400 (78.6) | 7491 (79.1) | 2685 (25.3) | 31 033 (57.6) | 22 013 (93.7) | 30 234 (72.3) | 10 119 (55.6) |
| Wealth quintile | Poorest | 68 822 (26.9) | 30 444 (30.9) | 3106 (32.8) | 2326 (21.9) | 12 067 (22.4) | 7272 (31.0) | 9054 (21.6) | 4553 (25.0) |
|  | Poorer | 54 248 (21.2) | 20 339 (20.6) | 2133 (22.5) | 2229 (21.0) | 10 893 (20.2) | 6316 (26.9) | 8834 (21.1) | 3504 (19.2) |
|  | Middle | 49 211 (19.2) | 18 014 (18.3) | 1680 (17.7) | 2416 (22.8) | 10 290 (19.1) | 4800 (20.4) | 8645 (20.7) | 3366 (18.5) |
|  | Richer | 44 555 (17.4) | 15 789 (16.0) | 1400 (14.8) | 2253 (21.2) | 10 053 (18.7) | 3147 (13.4) | 8717 (20.8) | 3196 (17.6) |
|  | Richest | 39 195 (15.3) | 13 946 (14.2) | 1155 (12.2) | 1379 (13.0) | 10 586 (19.6) | 1960 (8.3) | 6573 (15.7) | 3596 (19.7) |
| Urban or rural | Urban | 64 275 (25.1) | 27 796 (28.2) | 2805 (29.6) | 4652 (43.9) | 10 074 (18.7) | 7005 (29.8) | 8692 (20.8) | 3251 (17.8) |
| TOTAL N  (% of total N) | | 256,031 | 98 532 (38.4) | 9474 (3.7) | 10 604 (4.1) | 53 889 (21.0) | 23 495 (9.2) | 41 823 (16.3) | 18 215 (7.1) |
| <1 year Deaths  N (%) | | 13,629 (5.3) | 4737 (4.8) | 562 (5.9) | 438 (4.1) | 3643 (6.8) | 953 (4.1) | 2182 (5.2) | 1114 (6.1) |
| Age 1-5 Deaths  N (%) | | 5149  (2.0) | 1737 (1.8) | 259 (2.7) | 103 (1.0) | 1528 (2.8) | 258 (1.1) | 795 (1.9) | 469 (2.6) |
