# Supplementary material for: Effect of health systems context on infant and child mortality in sub-Saharan Africa from 1995 to 2015, a longitudinal cohort analysis
Source: Sci Rep. 2021 Aug 11;11:16263. doi: 10.1038/s41598-021-95886-8 (PMC8357794; doi:10.1038/s41598-021-95886-8)
Supplement: Supplementary file 4 — Supplementary Table S3. [file 41598_2021_95886_MOESM4_ESM.docx]

Table S3: Full model results (acceleration factors and associated 95% confidence intervals) from adjusted accelerated failure time models for child and infant survival, results including covariates at the child, mother and household level

|  | | | |  |  |  |  |
| --- | --- | --- | --- | --- | --- | --- | --- |
|  |  |  |  |  | | | |
|  | **CHILD SURVIVAL** | | **INFANT SURVIVAL** | | | | |
| **Covariate** |  | **Acceleration factor (95% Confidence Intervals)** |  | **Acceleration factor (95% Confidence Intervals)** | | | |
| **Region level health systems factors** | | |  |  | | | |
| Ratio facilities to population |  | 0.97 (0.94, 0.99) |  | 1.02 (0.99, 1.04) | | | |
| Proportion private facilities |  | 0.81 (0.79, 0.84) |  | 0.87 (0.85, 0.9) | | | |
| Proportion with a doctor |  | 1.11 (1.08, 1.14) |  | 1.03 (1.01, 1.05) | | | |
| Proportion trained in IMCI |  | 0.99 (0.96, 1.02) |  | 1.01 (0.98, 1.03) | | | |
| Proportion charging fees for sick child services |  | 0.98 (0.88, 1.1) |  | 1.03 (0.94, 1.14) | | | |
| Proportion charging fees for immunization |  | 1.20 (1.12, 1.28) |  | 0.96 (0.9, 1.01) | | | |
| Proportion charging fees for delivery |  | 0.82 (0.74, 0.91) |  | 1.11 (1.01, 1.21) | | | |
| **Child-level covariates** | | |  |  | | | |
| Male |  | 1.04 (1, 1.09) |  | 1.28 (1.23, 1.33) | | | |
| First birth |  | 1 (Reference) |  | 1 (Reference) | | | |
| Birth order (X) |  | 1.08 (1.02, 1.15) |  | 0.85 (0.81, 0.9) | | | |
| Birth order (X) |  | 1.31 (1.19, 1.43) |  | 1.04 (0.97, 1.13) | | | |
| Birth year |  | 0.79 (0.77, 0.82) |  | 0.8 (0.78, 0.82) | | | |
| **Mother-level covariates** | | |  |  | | | |
| Maternal age |  | 0.89 (0.85, 0.93) |  | 0.99 (0.95, 1.02) | | | |
| Maternal education: none |  | 1 (Reference) |  | 1 (Reference) | | | |
| Maternal education: Primary |  | 0.85 (0.8, 0.89) |  | 0.84 (0.8, 0.88) | | | |
| Maternal education: Secondary or higher |  | 0.65 (0.57, 0.73) |  | 0.7 (0.64, 0.76) | | | |
| Married |  | 0.78 (0.74, 0.82) |  | 0.82 (0.79, 0.86) | | | |
| **Household-level covariates** | | |  |  | | | |
| Wealth quintile: 1 |  | 1.08 (1.01, 1.15) |  | 1.02 (0.97, 1.07) | | | |
| Wealth quintile: 2 |  | 0.96 (0.9, 1.03) |  | 1.01 (0.95, 1.07) | | | |
| Wealth quintile: 3 |  | 0.92 (0.86, 0.99) |  | 1 (0.94, 1.06) | | | |
| Wealth quintile: 4 |  | 0.73 (0.66, 0.81) |  | 0.89 (0.83, 0.97) | | | |
| Wealth quintile: 5 |  | 1 (Reference) |  | 1 (Reference) | | | |
| Urban |  | 0.96 (0.9, 1.02) |  | 1.01 (0.96, 1.07) | | | |
| **Country fixed-effects** | | |  |  | | | |
| Tanzania |  | 1 (Reference) |  | 1 (Reference) | | | |
| Kenya |  | 0.82 (0.73, 0.92) |  | 0.83 (0.76, 0.92) | | | |
| Ghana |  | 1.15 (0.99, 1.32) |  | 0.91 (0.8, 1.03) | | | |
| Namibia |  | 0.87 (0.72, 1.05) |  | 0.8 (0.69, 0.93) | | | |
| Rwanda |  | 1.59 (1.38, 1.84) |  | 1.06 (0.94, 1.2) | | | |
| Senegal |  | 1.00 (0.85, 1.18) |  | 0.88 (0.78, 1.01) | | | |
| Uganda |  | 1.88 (1.68, 2.10) |  | 1.21 (1.1, 1.33) | | | |
|  |  |  |  |  | | | |
|  |  |  |  |  | | | |
