# Supplementary material for: Effect of health systems context on infant and child mortality in sub-Saharan Africa from 1995 to 2015, a longitudinal cohort analysis
Source: Sci Rep. 2021 Aug 11;11:16263. doi: 10.1038/s41598-021-95886-8 (PMC8357794; doi:10.1038/s41598-021-95886-8)
Supplement: Supplementary file 5 — Supplementary Table S4. [file 41598_2021_95886_MOESM5_ESM.docx]

| **Region-level health systems factors** | Ratio facilities to population | Proportion private facilities | Proportion with a doctor | Proportion trained in IMCI | Proportion charging fees for sick child services | Proportion charging fees for immunization | Proportion charging fees for delivery |
| --- | --- | --- | --- | --- | --- | --- | --- |
| Ratio facilities to population | 1 |  |  |  |  |  |  |
| Proportion private facilities | -0.07  (-0.23, 0.09) | 1 |  |  |  |  |  |
| Proportion with a doctor | -0.15  (-0.30, 0.01) | 0.26  (0.10, 0.40) | 1 |  |  |  |  |
| Proportion trained in IMCI | -0.08  (-0.24, 0.08) | -0.14  (-0.29, 0.02) | -0.27  (-0.41, -0.11) | 1 |  |  |  |
| Proportion charging fees for sick child services | -0.01  (-0.17, 0.15) | 0.33  (0.18, 0.47) | 0.12  (-0.04, 0.28) | -0.13  (-0.28, 0.04) | 1 |  |  |
| Proportion charging fees for immunization | -0.01  (-0.17, 0.15) | 0.23  (0.07, 0.37) | 0.20  (0.04, 0.35) | 0.04  (-0.12, 0.20) | 0.52  (0.39, 0.63) | 1 |  |
| Proportion charging fees for delivery | -0.13  (-0.28, 0.03) | 0.47  (0.34, 0.59) | 0.10  (-0.07, 0.25) | -0.12  (-0.27, 0.05) | 0.65  (0.55, 0.74) | 0.46  (0.33, 0.58) | 1 |

Table S4. Spearman correlation matrix (with 95% confidence intervals) for region-level health systems factors included in the accelerated failure time models.
