# Supplementary material for: Effect of health systems context on infant and child mortality in sub-Saharan Africa from 1995 to 2015, a longitudinal cohort analysis
Source: Sci Rep. 2021 Aug 11;11:16263. doi: 10.1038/s41598-021-95886-8 (PMC8357794; doi:10.1038/s41598-021-95886-8)
Supplement: Supplementary file 6 — Supplementary Table S5. [file 41598_2021_95886_MOESM6_ESM.docx]

Table S5: Unadjusted and adjusted estimated hazard ratios (95% Confidence interval) for the effect of health system characteristics on time to any death from a child’s birth to their fifth birthday. These estimates are exponentiated coefficients from an accelerated failure time model and approximate the hazard ratio.

|  |  | **ALL SURVIVAL TO FIFTH BIRTHDAY** | |
| --- | --- | --- | --- |
|  | **Covariate** | **Unadjusted** | **Adjusted^1^** |
|  | **Region level health systems factors** | | |
| **Access** | Ratio facilities to population^2^ | 1.00 (0.98, 1.03) | 1.01 (0.98, 1.04) |
|  | Proportion private facilities | 0.76 (0.74, 0.78) | 0.83 (0.77, 0.81) |
| **Quality** | Proportion with a doctor | 1.07 (1.05, 1.1) | 1.07 (1.04, 1.1) |
|  | Proportion trained in IMCI^3^ | 0.96 (0.93, 0.99) | 0.99 (0.97, 1.02) |
| **Cost** | Proportion charging fees for sick child services^4^ | 0.87 (0.79, 0.96) | 1.07 (0.97, 1.18) |
|  | Proportion charging fees for immunization^4^ | 1.18 (1.11, 1.25) | 1.09 (1.02, 1.16) |
|  | Proportion charging fees for delivery^4^ | 0.97 (0.88, 1.06) | 0.98 (0.89, 1.08) |

^1^Adjusted for child-level (male, birth order and birth year), maternal-level (maternal age, education and marital status) and household-level (urban, wealth score) factors

^2^The ratio of facilities in a region divided by the ratio of the population in the same region

^3^Proportion of health staff trained in Integrated Management of Childhood Illness per facility averaged across all facilities in the region

^4^Proportion of facilities in a region charging any fees for these types of services, including for consultation, registration, health card, drugs, vaccines, syringes or needles
